# An Inverse Correlation between the Atherogenic Index of Plasma and Heart Failure: An Analysis of the National Health and Nutrition Examination Survey 2017–March 2020 Pre-Pandemic Data

**DOI:** 10.3390/jcdd9120412

**Published:** 2022-11-23

**Authors:** Jianying Xue, Lu He, Hang Xie, Xuegang Xie, Haiyan Wang

**Affiliations:** Department of Structural Heart Disease, The First Affiliated Hospital of Xi’an Jiaotong University, Xi’an 710061, China

**Keywords:** atherogenic index of plasma, heart failure, NHANES, cardiovascular disease

## Abstract

Aims: The atherogenic index of plasma (AIP) is associated with cardiovascular diseases. Nevertheless, limited studies have investigated the association between AIP and the risk of heart failure (HF) in the general population. This study aimed to explore the association between AIP and HF risk using a large-scale population dataset from the National Health and Nutrition Examination Survey (NHANES) 2017–March 2020 Pre-pandemic data. Methods: A total of 5598 individuals were included in the analysis of the association between AIP and HF from the NHANES database. The relationship between AIP and HF was examined using multivariate logistic regression and smooth curve fitting. An association between the two was detected based on the odds ratios (ORs) and 95% confidence intervals (CIs). Results: The overall prevalence of HF among the 5598 participants analyzed was 3.21%. Compared with individuals in the lowest quartile of AIP, participants in the higher quartiles showed a significantly reduced probability of HF. Smooth curve fitting analysis revealed a linear association between AIP and HF. Compared with individuals in Q1 of the AIP, participants in Q2 (OR = 0.38, 95% CI = 0.2–0.72, *p* = 0.0033), Q3 (OR = 0.24, 95% CI = 0.12–0.48, *p* < 0.0001), and Q4 (OR = 0.32, 95% CI = 0.14–0.74, *p* = 0.0075) had a significantly decreased risk of HF after adjusting for other risk factors. Analysis of subgroup strata revealed that AIP may interact with age and statin use (*p* for interaction = 0.012 and 0.0022, respectively). Conclusion: Our results suggest that a high AIP value is negatively correlated with HF prevalence. The AIP may be an effective method for identifying individuals at a high risk of HF.

## 1. Introduction

Heart failure (HF) is clinically characterized by impaired cardiac structure and function, quality of life, and altered neurohormonal regulation [1]. HF is a manifestation of a late stage in various heart diseases. HF affects an estimated 64.3 million people globally [2]. In the United States, a study based on the National Inpatient Sample found an increase in the number of hospitalization cases from 1,060,540 in 2008 to 1,270,360 in 2018 [3]. According to the American Heart Association, the prevalence of HF continues to rise, resulting in increased economic and social costs [4]. As the population age and cardiovascular diseases increase, the prevalence of HF is expected to continuously rise in the future. Therefore, to combat this growing trend, novel preventive measures targeting key risk factors are required urgently.

There are several etiologies of HF, such as coronary artery disease, rheumatic heart disease, cardiomyopathy, congenital heart disease, high blood pressure, and hyperthyroidism [5]. Among these diseases, ischemic HF is common. In 2010, North America, Oceania, and Eastern Europe had the highest prevalence of ischemic heart failure (>5 per 1000) [6]. Clinical trials and epidemiological studies indicate that patients with ischemic HF have a worse prognosis than those with non-ischemic HF [7]. A growing body of evidence suggests that unfavorable blood glucose and cholesterol levels are major risk factors for HF. There is a close relationship between blood lipid levels and HF syndrome [8]. Recently, the plasma lipid profile has been identified as an important risk factor and predictor of cardiovascular disease [9]. The atherogenic index of plasma (AIP), calculated using the formula of log (triglyceride/high-density lipoprotein cholesterol) [9], is a new index composed of triglycerides (TG) and high-density lipoprotein cholesterol (HDL-C) levels. AIP might have the potential of becoming HF biomarker. AIP not only accurately represents the true relationship between protective and atherogenic lipoproteins but also serves as a strong predictor of atherosclerosis and coronary heart disease [10]. However, to date, no study has investigated the association between AIP and HF. Therefore, we aimed to investigate the association between AIP and HF incidence in a large nationally representative sample of the U.S. population. We further explored the interactions and stratified confounders in the association of AIP and HF in different subgroups.

## 2. Materials and Methods

### 2.1. Study Population

The NHANES database is an ongoing cross-sectional nationally representative survey conducted by the National Center for Health Statistics (NCHS) to assess the health status of US residents. It is designed to supervise the health and nutritional status of civilian, non-institutionalized US inhabitants using a complex, multistage design with data released in two-year cycles. Details of the NHANES study design and data can be accessed at http://www.cdc.gov/nchs/nhanes.htm (accessed on 4 September 2022). The baseline demographic and health-related questions were collected through in-person interviews. In addition to home interviews, NHANES participants underwent health assessments at a mobile examination center (MEC), clinical examinations, and laboratory investigations. NHANES field operations were suspended in March 2020 due to the coronavirus disease 2019 (COVID-19) pandemic. For this reason, NHANES 2019–March 2020 cycle data were combined with the data collected from the NHANES 2017–2018 cycle and a national representative sample was created from NHANES 2017–March 2020 pre-pandemic data. 

**Figure 1:**
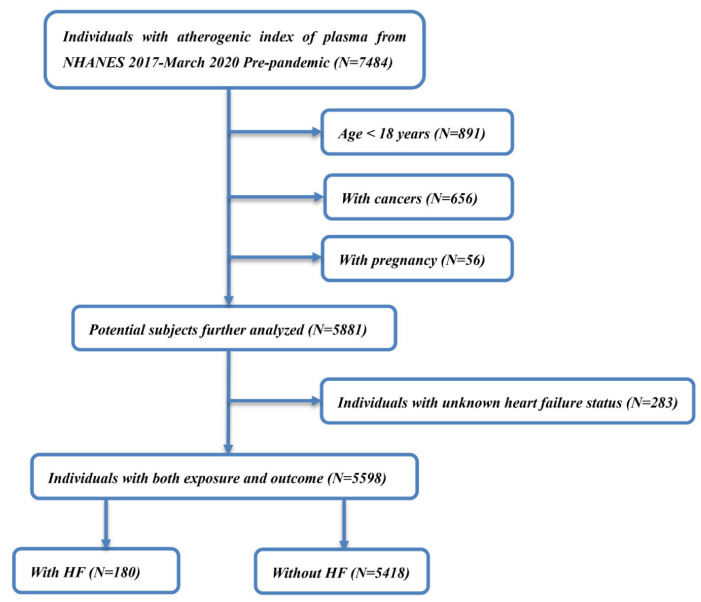
A detailed flowchart of study population selection.

The present study analyzed the data of 7484 participants with AIP available from the NHANES 2017–March 2020 pre-pandemic. Figure 1 shows a flowchart of the study subject selection from the NHANES database. Subjects were excluded if they were younger than 18 years (*n* = 891), had a diagnostic history of cancer (*n* = 656), or were pregnant at the time of examination (*n* = 56). Participants with no information on their HF status were also excluded (*n* = 283). Finally, 5,598 eligible participants were included in the analysis. This study conforms to the ethical guidelines of the 1975 Declaration of Helsinki. This study used public data from the NHANES, which was approved by the Institutional Review Board (IRB).

### 2.2. Definitions of HF and AIP

Similar to previous studies, the incident of HF was based on self-reported “Yes” to the MCQ questionnaire by asking the question, “Has a doctor or other health professional ever told you that you had congestive heart failure?” [11]. AIP is mathematically derived from log10 (TG/HDL-C), which is a logarithmic relationship between TG and HDL-C [9]. Subsequently, all participants were classified into four groups according to their AIP quartiles.

### 2.3. Covariates

Demographic information and characteristics such as gender, age, race, waist circumference, education level, marital status, smoking history, stroke, coronary heart disease, heart attack (also called as myocardial infarction), angina, statin use, diabetes medication, and antihypertensive medication were obtained using standardized household questionnaires. Races were categorized as Mexican American, non-Hispanic white, non-Hispanic black, or others. Education levels were grouped into pre-high school, high school, and above high school. Marital status was divided into two categories, i.e., unmarried (never married/divorced/separated/widowed) and married (married/living with a partner). Smokers were classified into three categories: never, former, and current smokers. The participants measured their height and weight while wearing light clothing and no shoes. Body mass index (BMI) was calculated as weight (kg) divided by height squared (m). Based on the BMI, participants were divided into three groups: normal (18.5 < BMI < 25 kg/m^2^), overweight (25 ≤ BMI ≤ 30 kg/m^2^), and obese (BMI > 30 kg/m^2^) 

The estimated glomerular filtration rate (eGFR) was calculated using the Chronic Kidney Disease Epidemiology Collaboration (CKD-EPI) equation [12] and was grouped into three categories <60 mL/min/1.73 m^2^, 60–90 mL/min/1.73 m^2^, and ≥90 mL/min/1.73 m^2^. Diabetes was defined as self-reported physician-diagnosed diabetes, medication to lower blood glucose or HbA1c level of less than 6.5%. A history of hypertension was defined as a self-reported hypertension diagnosis, diastolic blood pressure ≥90 mmHg, systolic blood pressure ≥140 mmHg or use of anti-hypertensive medication. MetS was defined according to the 2009 joint statement of the International Diabetes Federation (IDF) [13]. The selection of other laboratory test indicators was based on the previous literature, which included glucose (mg/dL), HbA1c (%), aspartate aminotransferase (AST), alanine aminotransferase (ALT), total protein (g/L), albumin (g/L), globulin (g/L), creatinine (mg/dL), uric acid (mg/dL), blood urea nitrogen (mg/dL), triglyceride (mg/dL), total cholesterol (mg/dL), HDL cholesterol (mg/dL), LDL cholesterol (mg/dL), and high-sensitivity C-reactive protein (hs-CRP). A detailed description of testing procedures and quality control strategies can be found on the NHANES webpage (https://www.cdc.gov/nchs/nhanes/ (accessed on 4 September 2022)).

### 2.4. Statistical Analysis

Data analysis was performed using EmpowerStats version 4.1 and R version 4.0.3 software. Since the NHANES used a complex multistage sample design, statistical analysis was performed using appropriate NHANES sampling weights. Continuous variables were expressed as a survey-weighted mean (95% CI) and categorical variables were presented as a survey-weighted percentage (95% CI). All individuals were divided into four groups according to the quartiles of the AIP level: Q1 (<−0.34), Q2 (−0.34 to −0.12), Q3 (−0.12 to 0.1), and Q4 (≥0.1). The first quintile was used as a reference group. Analysis of the trend between the quartiles was performed using a general linear model. The association between AIP and HF was examined using logistic regression analysis. Variables with more than 10% missing values were excluded from the model. The variance inflation factor (VIF) was used to check for multicollinearity, and variables with a VIF greater than 5 were excluded. Covariates were selected as potential confounders in the final models if they changed the AIP estimates on HF risk by more than 10% or were notably associated with the HF [14]. The multivariate logistic regression models included the unadjusted model, minimally adjusted model 1 (adjusted for gender, age, BMI, education level, and smoking), and a fully adjusted model 2 (adjusted for gender, age, BMI, education level, smoking, coronary heart disease, heart attack, angina, stroke, MetS, diabetes, eGFR, hypertension, glucose, HbA1c, ALT, albumin, globulin, creatinine, blood urea nitrogen, HDL cholesterol, hs-CRP, statin use, diabetes medication, and antihypertensive medication). We also performed additional analyses using AIP as a continuous variable. The association between the two was assessed by logistic regression using the same models. We also investigated the nonlinear dose–response relationship between the AIP and incident HF using smooth curve fitting. To test for interactions, we performed the stratified analyses using the Wald test. A significant interaction *p*-value indicates a population with special characteristics. A non-significant interaction *p*-value implies that the different levels of analysis are consistent and reliable. Stratified analyses according to gender (female and male), age (<60 years and ≥60 years), coronary heart disease (yes and no), myocardial infarction (yes and no), MetS (yes and no), smoking (never, former, now), eGFR (<60 mL/min/1.73 m^2^, 60–90 mL/min/1.73 m^2^, and ≥90 mL/min/1.73 m^2^), BMI (<25 kg/m^2^, 25–30 kg/m^2^, and ≥30 kg/m^2^), and statin use (yes and no) were performed to explore potential modifying effects. 

Sensitivity analyses were performed to test the robustness of the results. First, multiple imputations of missing values were analyzed ten times. The estimates from each model were combined by the Rubin rule using the pool() function of the mice package. Secondly, as the presence of unmeasured confounding factors in observational epidemiology is inevitable, a sensitivity analysis using the E-value algorithm was employed to address the possible effect of unmeasured confounding on the primary results. The E-value represents the minimum strength at which a confounder needs to be associated with both HF and AIP to fully explain their observed association [15].

## 3. Results

### 3.1. Baseline Characteristics of Included Individuals

A total of 5598 participants were enrolled in the study. Of these, 180 (3.21%) were diagnosed with HF. The weight demographics of the participants as per their AIP quartile are shown in Table 1. AIP was associated with age, gender, BMI, waist circumference, race/ethnicity, education level, smoking, fasting blood glucose, HbA1c, ALT, albumin, globulin, uric acid, triglyceride, total cholesterol, HDL cholesterol, LDL cholesterol, hs-CRP, coronary heart disease, heart attack, angina, MetS, diabetes, hypertension, statin use, and diabetes medication (all *p* < 0.05).

### 3.2. AIP and the Risk of HF

**Figure 2:**
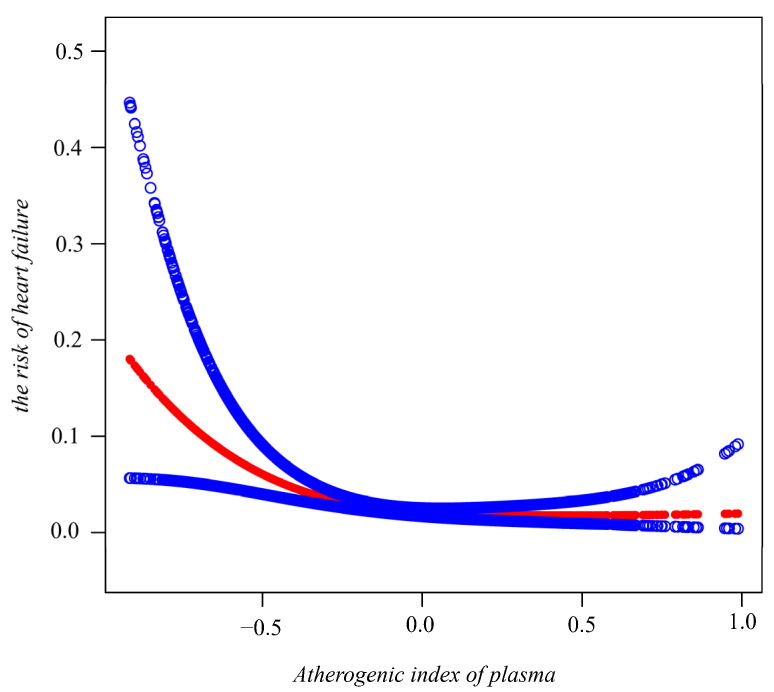
Association between the atherogenic index of plasma and incident heart failure using smooth curve fitting analysis. Adjusted for gender, age, BMI, education level, smoke, coronary heart disease, heart attack, angina, stroke, MetS, diabetes, eGFR, hypertension, glucose, HbA1c, ALT, albumin, globulin, creatinine, blood urea nitrogen, HDL cholesterol, hs-CRP, statins use, diabetes medication, and antihypertensive medication. The red and blue lines demonstrate the estimated values and their corresponding 95% CIs, respectively.

On the whole, a smooth curve fitting demonstrated a downward trend between AIP and HF prevalence. From the diagram, it can be seen that the slope in the first quarter is steep, while in the remaining quarters it continues to descend, i.e., the incident HF decreased relatively rapidly and subsequently became steady, gradually (Figure 2). Table 2 shows the crude and fully adjusted associations between AIP and incident HF. In the fully adjusted model, individuals in Q1 were used as references and participants in the highest quartile (Q4) showed a reduced risk of HF (OR = 0.32, 95% CI = 0.14–0.74; *p* = 0.0075). Further, individuals in Q2 (OR = 0.38, 95% CI = 0.20–0.72; *p* = 0.0033) and Q3 (OR = 0.24, 95% CI = 0.12–0.48; *p* < 0.0001) displayed an inverse relationship between AIP and HF. When using AIP as a continuous variable, the results were unchanged. In the fully adjusted model, after controlling for confounders, each unit of increased AIP was associated with a 72% decreased risk of HF (aHR = 0.28; 95% CI: 0.10–0.78; *p* = 0.0154).

### 3.3. Stratified Analysis and Sensitivity Analyses

**Figure 3:**
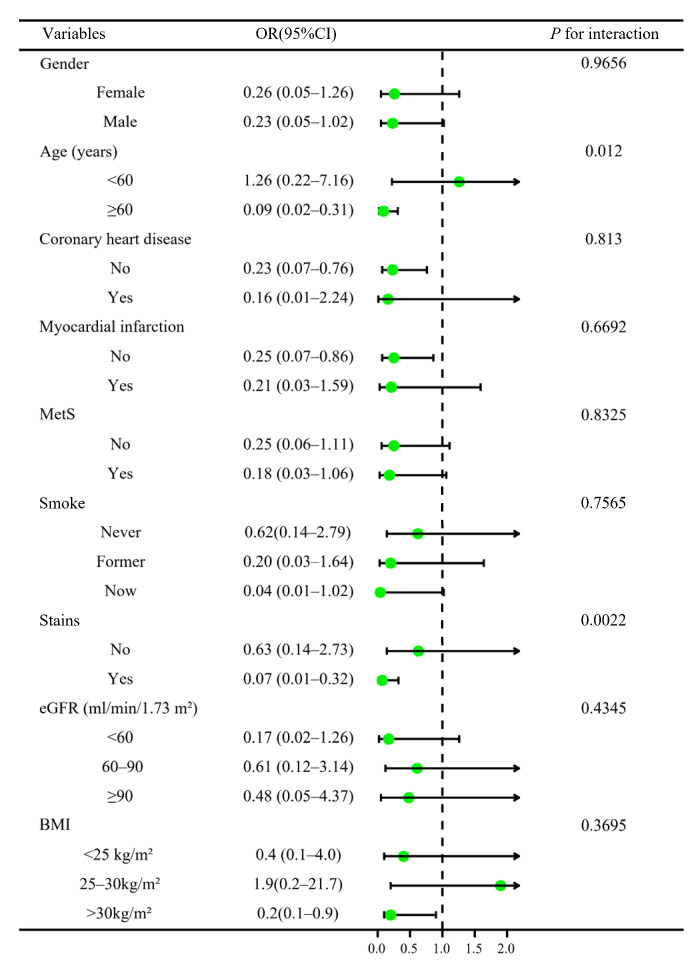
Forest plots of stratified analyses of atherogenic index of plasma and incident heart failure. Age, gender, BMI, education level, smoke, coronary heart disease, heart attack, angina, stroke, MetS, diabetes, eGFR, hypertension, glucose, HbA1c, ALT, albumin, globulin, creatinine, blood urea nitrogen, HDL cholesterol, hs-CRP, statins use, diabetes medication, and antihypertensive medication were all adjusted except the stratification variable itself.

For a more detailed analysis of the association between AIP and HF, we divided patients based on their demographic characteristics. Stratified analyses were conducted in different subgroups to identify interactions and confounders that may affect the association between AIP and HF (Figure 3). Results indicated that age (<60 and ≥60 years) had different effects on the association between AIP and HF (*p* for interaction = 0.012). The inverse association between AIP and incident HF was more pronounced in individuals over 60 years of age. At the same time, statin use also appeared to interact with the association between AIP and HF (*p* for interaction = 0.0022). 

Sensitivity analyses using 10 rounds of multiple imputation data did not significantly alter the main findings (Figure S1). Using AIP as a continuous variable, the association between AIP and incident HF remained significant when combined with 10 rounds of multiple imputations into the final estimates (OR = 0.37, 95% CI = 0.14–0.98, *p* = 0.0485). After full adjustment, compared with individuals in Q1, AIP was still inversely associated with HF in Q2 (OR = 0.44, 95% CI = 0.24–0.82, *p* = 0.0099), Q3 (OR = 0.27, 95% CI = 0.13–0.54, *p* < 0.001), and Q4 (OR = 0.44, 95% CI = 0.19–0.99, *p* = 0.032). Furthermore, the E-value (and its lower limit of 95% CI) for the relationship between AIP and the prevalence of HF was 3.19 (1.52) (Figure S2). The result can be interpreted as unmeasured confounders with 1.52-fold risk ratios associated with both AIP and HF, respectively, outweighed measured confounders, but weaker confounders did not. Therefore, the E-value provided evidence that the study was robust.

## 4. Discussion

In this large-sample cross-sectional study, the results indicate that after adjusting for multiple relevant confounders, individuals with high AIP levels had an adverse association with HF. Therefore, maintaining a high AIP may help in reducing the risk of HF. To the best of our knowledge, this is the first study to demonstrate a relationship between AIP and incident HF in a large representative sample of US adults. Our results fill an information gap on the association of AIP and incident HF by demonstrating an inverse relationship.

AIP, calculated as log10 (TG/HDL-C), was originally developed as a biomarker of plasma atherosclerosis and is now being used as a predictive index for coronary artery disease [16,17]. AIP has also been proposed as a clinical indicator of cardiovascular and metabolic disorders [18]. There is evidence that dyslipidemia is a major risk factor for coronary artery disease [19]. After adjusting for multiple traditional cardiovascular risk factors, AIP was found to be significantly associated with cardiovascular risk. This could be an effective method for selecting individuals at high cardiovascular risk [20]. Multiple causes contribute to HF, leading to high all-cause mortality in hospitals. Therefore, it is important to identify modifiable risk factors from a public health perspective to prevent HF. As an important risk factor for HF, modifying lipids can reduce the risk of HF [21].

AIP can be used as a marker for the presence of small dense LDL (sd-LDL) [16]. An increase in AIP is correlated with larger LDL particles, making it an ideal indicator of atherogenic lipoproteins. Dobiosova first defined the AIP in 2001 and suggested that it could be used as a biomarker for plasma atherogenicity due to its relationship with LDL-C particle size [16]. The sd-LDL invades the arterial wall and forms deposits more readily, it is more sensitive to oxidative stress and is readily oxidized to LDL. Upon phagocytosis by macrophages, oxidized LDL becomes foam cells thereby worsening atherosclerosis [22]. Atherogenic lipoproteins, with smaller particle sizes, migrate more easily and are oxidized, thereby accelerating atherosclerosis that exacerbates coronary artery disease [23]. Previous studies have found an association between AIP and small, dense lipoprotein particles, which can be used as a marker of atherogenicity [24]. In HF patients, HDL-C was significantly lower and TG levels were significantly higher than in controls [22]. Increased inflammatory conditions and decreased HDL-C levels in HF suggest that dyslipidemia has an atherosclerotic effect [25]. HDL-C has antithrombotic, anti-inflammatory, antioxidant, and antiatherogenic effects. HDL may reduce thrombus formation by reducing platelet reactivity and aggregability. Furthermore, previous studies have demonstrated that AIP is positively associated with TG, TC, and LDL-C, and negatively with HDL-C [26,27,28]. Furthermore, our stratified analyses identified a specific population where individuals in different age groups (<60 and ≥60 years) and with statin use contribute differently to the association between AIP and HF. AIP not only accurately represents the link between protective and atherogenic lipoproteins but also acts as a powerful predictor of atherosclerosis and CAD [10]. AIP values below 0.11 are associated with low CVD risk, while those between 0.11 and 0.21 and above 0.21 are associated with intermediate and increased CVD risk [29]. In most previous studies, the AIP was compared between patients with overt CAD and controls. However, the results were inconsistent [30,31]. Thus, further studies with a larger number of participants are warranted to explore the detailed mechanisms that a high AIP value is negatively correlated with HF prevalence.

This study had some limitations. First, the findings of this study may not be applicable to the other ethnic groups because we included individuals from the US only. Thus, the findings of this study need to be validated in other populations. Second, we were unable to compare the association between the AIP and the incidence of HF in different heart failure subgroups since the ejection fraction was unavailable in NHANES. Third, despite adjusting for most demographic and clinical variables, the possibility of unmeasured confounding remains. However, we obtained robust results via multiple sensitivity analyses and the evaluation of E-values. These results support the robustness of our conclusions. Besides, the inclusion of variables is based on the standard that introducing covariates in the basic model or eliminating covariates in the final model has a more than 10% influence on the regression coefficient of AIP, not the “10 Events Per Variable (EPV)” criterion. This may also have some influence on the results.

## 5. Conclusions

The current investigation showed that a high AIP value is negatively correlated with the prevalence of HF. The AIP can be an effective method for identifying individuals at high risk of HF. Further studies are required to determine whether interfering with AIP will reduce incident HF in clinical practice.

## Figures and Tables

**Table 1:** Baseline characteristics of included individuals according to atherogenic index of plasma quartiles, weighted.

	Q1	Q2	Q3	Q4	*p*-Value
Age (years)	43.07 (41.14, 44.99)	45.93 (44.20, 47.65)	47.51 (45.49, 49.53)	48.05 (46.06, 50.04)	0.0202
BMI (kg/m^2^)	25.98 (25.32, 26.65)	28.70 (27.48, 29.93)	31.20 (30.58, 31.81)	32.29 (31.20, 33.38)	<0.0001
Waist circumference (cm)	90.09 (88.37, 91.81)	97.92 (95.22, 100.62)	104.21 (102.48, 105.94)	107.66 (105.18, 110.14)	<0.0001
eGFR (ml/min/1.73 m^2^)	99.51 (95.99, 103.04)	97.19 (94.74, 99.63)	95.55 (93.19, 97.92)	94.80 (92.21, 97.40)	0.2329
Glucose (mg/dl)	100.20 (99.33, 101.08)	104.51 (102.91, 106.10)	109.69 (106.68, 112.71)	123.53 (117.08, 129.98)	<0.0001
HbA1c (%)	5.35 (5.31, 5.39)	5.50 (5.44, 5.57)	5.72 (5.64, 5.80)	6.03 (5.87, 6.19)	<0.0001
ALT (IU/L)	20.17 (17.21, 23.12)	21.97 (19.88, 24.06)	23.72 (21.67, 25.77)	27.52 (26.14, 28.89)	0.0022
AST (IU/L)	23.29 (20.99, 25.59)	22.22 (20.53, 23.92)	21.78 (20.17, 23.40)	22.26 (21.59, 22.94)	0.7912
Total protein (g/L)	71.09 (70.23, 71.94)	70.72 (70.26, 71.19)	71.08 (70.69, 71.48)	70.80 (70.10, 71.50)	0.4635
Albumin (g/L)	41.27 (40.69, 41.85)	40.49 (40.06, 40.92)	39.98 (39.55, 40.41)	40.38 (39.82, 40.93)	0.0054
Globulin (g/L)	29.81 (29.10, 30.52)	30.23 (29.86, 30.61)	31.10 (30.75, 31.45)	30.42 (29.90, 30.95)	0.0033
Creatinine (mg/dl)	0.84 (0.82, 0.87)	0.86 (0.83, 0.89)	0.87 (0.85, 0.90)	0.90 (0.86, 0.95)	0.0952
Uric acid (mg/dl)	4.83 (4.69, 4.97)	5.32 (5.18, 5.47)	5.70 (5.54, 5.86)	5.96 (5.78, 6.14)	<0.0001
Blood urea nitrogen (mg/dl)	14.32 (13.70, 14.94)	13.82 (13.33, 14.32)	14.24 (13.76, 14.72)	15.22 (14.32, 16.11)	0.0778
Triglyceride (mg/dl)	49.28 (47.76, 50.79)	75.86 (72.84, 78.88)	109.00 (106.89, 111.10)	207.61 (191.35, 223.86)	<0.0001
Total cholesterol (mg/dl)	176.11 (171.32, 180.90)	182.52 (176.68, 188.35)	186.87 (181.56, 192.19)	199.78 (195.03, 204.54)	<0.0001
HDL cholesterol (mg/dl)	69.71 (67.70, 71.71)	55.98 (54.23, 57.74)	48.84 (47.75, 49.93)	41.09 (40.11, 42.07)	<0.0001
LDL cholesterol (mg/dl)	96.58 (92.56, 100.61)	111.39 (106.98, 115.80)	116.24 (111.91, 120.56)	119.13 (115.17, 123.09)	<0.0001
hs-CRP (mg/L)	2.31 (1.72, 2.90)	4.26 (3.04, 5.48)	4.26 (3.70, 4.81)	4.51 (3.57, 5.45)	0.0001
Sex					<0.0001
Female	60.76 (53.83, 67.29)	52.89 (45.27, 60.37)	47.27 (42.08, 52.51)	34.72 (26.26, 44.27)	
Male	39.24 (32.71, 46.17)	47.11 (39.63, 54.73)	52.73 (47.49, 57.92)	65.28 (55.73, 73.74)	
Race/ethnicity					<0.0001
Mexican American	6.84 (3.77, 12.10)	8.79 (4.69, 15.89)	11.53 (7.82, 16.68)	13.25 (9.09, 18.92)	
Non-Hispanic Black	17.37 (12.21, 24.11)	14.70 (11.47, 18.63)	9.71 (6.26, 14.76)	5.39 (3.17, 9.02)	
Non-Hispanic White	59.84 (52.59, 66.68)	59.81 (52.22, 66.95)	57.27 (52.48, 61.93)	61.76 (56.49, 66.78)	
Others	15.95 (11.85, 21.12)	16.70 (11.24, 24.11)	21.49 (16.81, 27.05)	19.60 (15.75, 24.11)	
Education level					<0.0001
Less Than High School	7.73 (5.14, 11.48)	9.52 (7.11, 12.63)	14.07 (11.02, 17.80)	17.26 (13.33, 22.06)	
High school or GED	25.05 (20.16, 30.67)	25.37 (19.67, 32.07)	32.66 (27.11, 38.75)	28.94 (24.26, 34.11)	
Above high school	67.22 (60.87, 72.99)	65.11 (57.20, 72.27)	53.26 (46.93, 59.49)	53.80 (47.14, 60.33)	
Marital status					0.0880
Unmarried	44.65 (36.10, 53.53)	38.87 (33.49, 44.53)	34.52 (27.61, 42.14)	35.02 (27.97, 42.79)	
Married	55.35 (46.47, 63.90)	61.13 (55.47, 66.51)	65.48 (57.86, 72.39)	64.98 (57.21, 72.03)	
Coronary heart disease					0.0289
No	97.46 (94.57, 98.83)	98.50 (96.79, 99.31)	97.10 (94.65, 98.45)	94.18 (87.05, 97.50)	
Yes	2.54 (1.17, 5.43)	1.50 (0.69, 3.21)	2.90 (1.55, 5.35)	5.82 (2.50, 12.95)	
Heart attach					0.0013
No	99.31 (98.03, 99.76)	97.01 (94.23, 98.48)	96.20 (92.97, 97.97)	94.37 (88.90, 97.23)	
Yes	0.69 (0.24, 1.97)	2.99 (1.52, 5.77)	3.80 (2.03, 7.03)	5.63 (2.77, 11.10)	
Angina					<0.0001
No	99.56 (98.59, 99.86)	98.79 (97.28, 99.46)	97.60 (95.23, 98.80)	93.86 (87.88, 96.99)	
Yes	0.44 (0.14, 1.41)	1.21 (0.54, 2.72)	2.40 (1.20, 4.77)	6.14 (3.01, 12.12)	
Stroke					0.4363
No	98.57 (96.80, 99.37)	97.51 (95.34, 98.69)	97.01 (94.57, 98.37)	97.40 (95.82, 98.39)	
Yes	1.43 (0.63, 3.20)	2.49 (1.31, 4.66)	2.99 (1.63, 5.43)	2.60 (1.61, 4.18)	
MetS					<0.0001
No	94.41 (90.13, 96.90)	89.10 (84.11, 92.66)	68.12 (60.91, 74.55)	33.62 (23.75, 45.17)	
Yes	5.59 (3.10, 9.87)	10.90 (7.34, 15.89)	31.88 (25.45, 39.09)	66.38 (54.83, 76.25)	
Smoke					0.0049
Never	62.15 (55.96, 67.97)	60.21 (51.53, 68.29)	58.19 (53.14, 63.07)	46.39 (39.26, 53.66)	
Former	25.77 (19.78, 32.84)	23.45 (17.57, 30.56)	22.18 (16.67, 28.87)	30.67 (25.98, 35.79)	
Now	12.08 (7.96, 17.91)	16.34 (12.81, 20.61)	19.63 (14.04, 26.76)	22.95 (16.96, 30.29)	
Diabetes					<0.0001
No	96.21 (94.89, 97.20)	93.59 (91.00, 95.47)	83.82 (79.51, 87.37)	72.91 (67.96, 77.36)	
Yes	3.79 (2.80, 5.11)	6.41 (4.53, 9.00)	16.18 (12.63, 20.49)	27.09 (22.64, 32.04)	
Hypertension					0.0004
No	71.77 (61.89, 79.92)	70.89 (63.22, 77.53)	55.24 (47.32, 62.90)	54.14 (45.93, 62.14)	
Yes	28.23 (20.08, 38.11)	29.11 (22.47, 36.78)	44.76 (37.10, 52.68)	45.86 (37.86, 54.07)	
Statins use					<0.0001
No	86.39 (80.93, 90.47)	89.58 (85.13, 92.81)	81.94 (78.13, 85.21)	75.97 (69.22, 81.64)	
Yes	13.61 (9.53, 19.07)	10.42 (7.19, 14.87)	18.06 (14.79, 21.87)	24.03 (18.36, 30.78)	
Antidiabetic drugs					<0.0001
No	97.10 (95.81, 98.01)	94.91 (92.84, 96.40)	86.35 (82.79, 89.26)	77.71 (72.23, 82.38)	
Yes	2.90 (1.99, 4.19)	5.09 (3.60, 7.16)	13.65 (10.74, 17.21)	22.29 (17.62, 27.77)	
Antihypertensive drugs					0.1399
No	98.05 (96.25, 99.00)	95.48 (90.46, 97.92)	95.14 (91.78, 97.17)	94.93 (92.20, 96.74)	
Yes	1.95 (1.00, 3.75)	4.52 (2.08, 9.54)	4.86 (2.83, 8.22)	5.07 (3.26, 7.80)	

Mean for continuous variables: The *p*-value was calculated by the weighted linear regression. Percent for categorical variables: *p*-value was calculated by weighted chi-square test.

**Table 2:** Associations between the atherogenic index of plasma and the risk of heart failure.

Exposure	Non-Adjusted OR (95%CI), *p*-Value	Adjust I OR (95%CI), *p*-Value	Adjust II OR (95%CI), *p*-Value
AIP	2.47 (1.57, 3.89) <0.0001	1.73 (1.00, 2.99) 0.0481	0.28 (0.10, 0.78) 0.0154
AIP quartile			
Q1	1(Reference)	1(Reference)	1(Reference)
Q2	1.18 (0.74, 1.87) 0.4937	0.99 (0.60, 1.62) 0.9648	0.38 (0.20, 0.72) 0.0033
Q3	1.03 (0.64, 1.65) 0.9173	0.64 (0.39, 1.07) 0.0879	0.24 (0.12, 0.48) <0.0001
Q4	2.16 (1.43, 3.27) 0.0003	1.41 (0.89, 2.24) 0.1433	0.32 (0.14, 0.74) 0.0075

Non-adjusted model adjusts for: None. Adjust I model adjust for: sex, age (continuous variable), BMI (continuous variable), education level, and smoke; Adjust II model adjust for: sex, age, BMI, education level, smoke, coronary heart disease, heart attack, angina, stroke, MetS, diabetes, eGFR, hypertension, glucose, HbA1c, ALT, albumin, globulin, creatinine, blood urea nitrogen, HDL cholesterol, hs-CRP, statins use, antidiabetic drug, and antihypertensive drug.

## Data Availability

Publicly available dataset was analyzed in this study. The National Health and Nutrition Examination Survey dataset are publicly available at https://www.cdc.gov/nchs/nhanes/index.htm (accessed on 4 September 2022).

## Supplementary Materials

The following supporting information can be downloaded at: https://www.mdpi.com/article/10.3390/jcdd9120412/s1, Figure S1: Pooled ORs for the atherogenic index of plasma and incident heart failure for multiple imputations (10 rounds) of missing data; Figure S2: E-value for the lower 95% CI, point estimate of the atherogenic index of plasma, and incident heart failure. The value of the joint minimum strength of association on the risk ratio scale that an unmeasured confounder must have with the exposure and outcome to fully describe the AIP and HF hazard ratios.
