# Supplementary material for: An Inverse Correlation between the Atherogenic Index of Plasma and Heart Failure: An Analysis of the National Health and Nutrition Examination Survey 2017–March 2020 Pre-Pandemic Data
Source: J Cardiovasc Dev Dis. 2022 Nov 23;9(12):412. doi: 10.3390/jcdd9120412 (PMC9786768; doi:10.3390/jcdd9120412)
Supplement: Supplementary file 1 [file jcdd-09-00412-s001.zip › jcdd-1981987-supplementary.pdf]

Multiple imputation	OR (95% CI)	pvalue
1(continuous)	0.37(0.14−0.98)	0.0448
Q1	1(Reference)	
Q2	0.44(0.24−0.82)	0.0099
Q3	0.27(0.13−0.54)	0.002
Q4	0.44(0.19−0.99)	0.0482
2(continuous)	0.38(0.14−1.01)	0.0522
Q1	1(Reference)	
Q2	0.45(0.24−0.83)	0.0115
Q3	0.27(0.14−0.55)	2e−04
Q4	0.46(0.20−1.04)	0.0626
3(continuous)	0.37(0.14−0.98)	0.0463
Q1	1(Reference)	
Q2	0.44(0.23−0.81)	0.0092
Q3	0.26(0.13−0.53)	2e−04
Q4	0.44(0.19−0.99)	0.0499
4(continuous)	0.36(0.13−0.96)	0.0415
Q1	1(Reference)	
Q2	0.43(0.23−0.81)	0.0084
Q3	0.26(0.13−0.52)	2e−04
Q4	0.43(0.19−0.98)	0.0437
5(continuous)	0.38(0.14−0.99)	0.0498
Q1	1(Reference)	
Q2	0.44(0.23−0.82)	0.0098
Q3	0.27(0.13−0.54)	2e−04
Q4	0.45(0.20−1.03)	0.0577
6(continuous)	0.37(0.14−0.99)	0.0499
Q1	1(Reference)	
Q2	0.44(0.24−0.82)	0.0102
Q3	0.27(0.13−0.54)	2e−04
Q4	0.44(0.19−1.01)	0.0514
7(continuous)	0.37(0.14−0.99)	0.0485
Q1	1(Reference)	
Q2	0.44(0.23−0.81)	0.0092
Q3	0.27(0.13−0.54)	2e−04
Q4	0.44(0.20−0.99)	0.0497
8(continuous)	0.38(0.14−0.99)	0.049
Q1	1(Reference)	
Q2	0.44(0.24−0.82)	0.0099
Q3	0.27(0.13−0.53)	2e−04
Q4	0.45(0.20−1.02)	0.0554
9(continuous)	0.38(0.15−1.01)	0.0534
Q1	1(Reference)	
Q2	0.44(0.24−0.83)	0.011
Q3	0.28(0.14−0.55)	3e−04
Q4	0.46(0.20−1.04)	0.0633
10(continuous)	0.37(0.14−0.99)	0.0481
Q1	1(Reference)	
Q2	0.44(0.24−0.83)	0.0104
Q3	0.27(0.14−0.55)	3e−04
Q4	0.45(0.20−1.01)	0.0536
Pooled(continuous)	0.37(0.14−0.98)	0.0485
Pooled(Q2 vs Q1)	0.44(0.24−0.82)	0.0099
Pooled(Q3 vs Q1)	0.27(0.13−0.54)	2e−04
Pooled(Q4 vs Q1)	0.44(0.19−0.99)	0.032

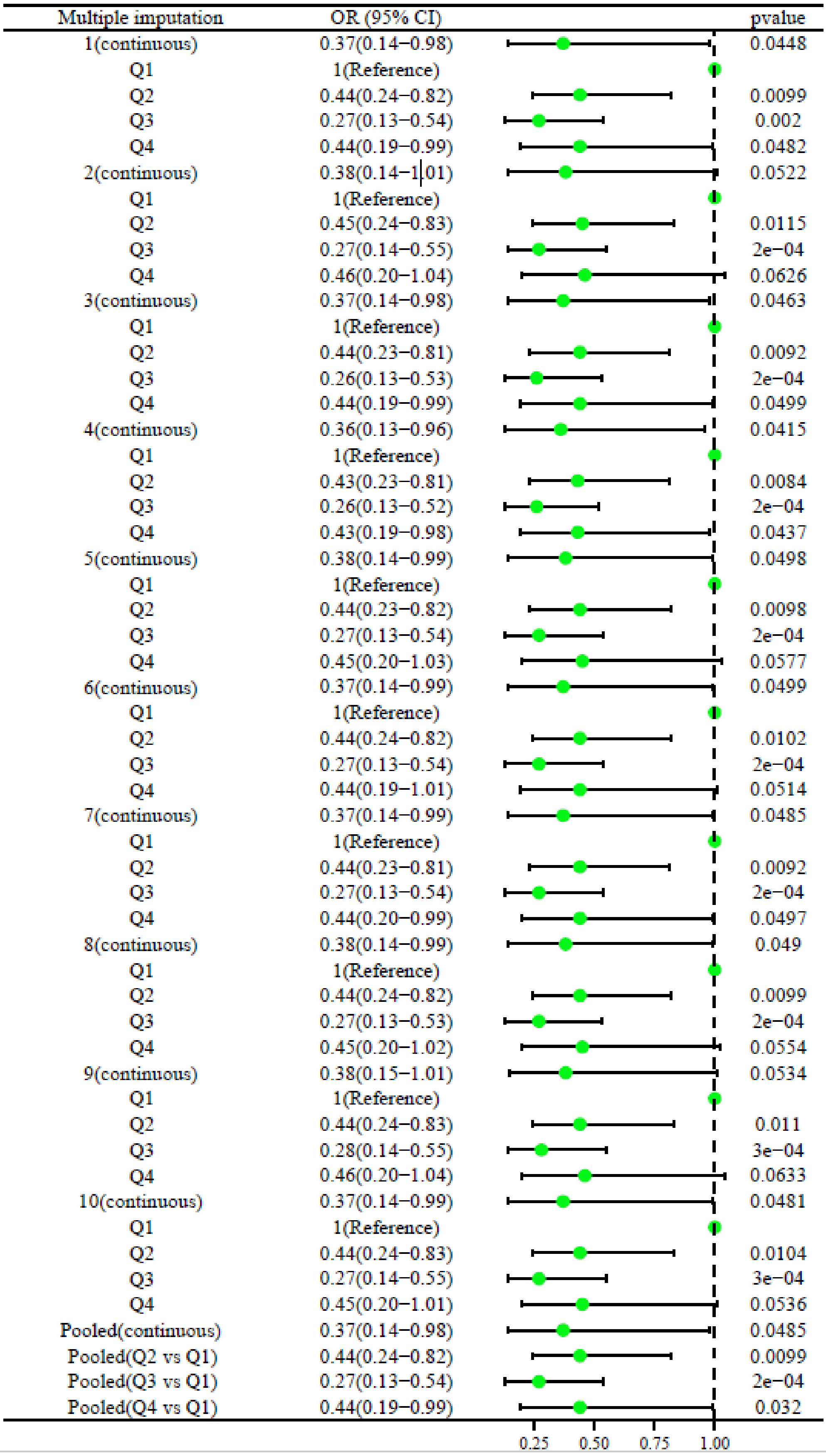

Figure S1: Pooled ORs for the atherogenic index of plasma and incident heart failure for multiple imputations (10 rounds) of missing data

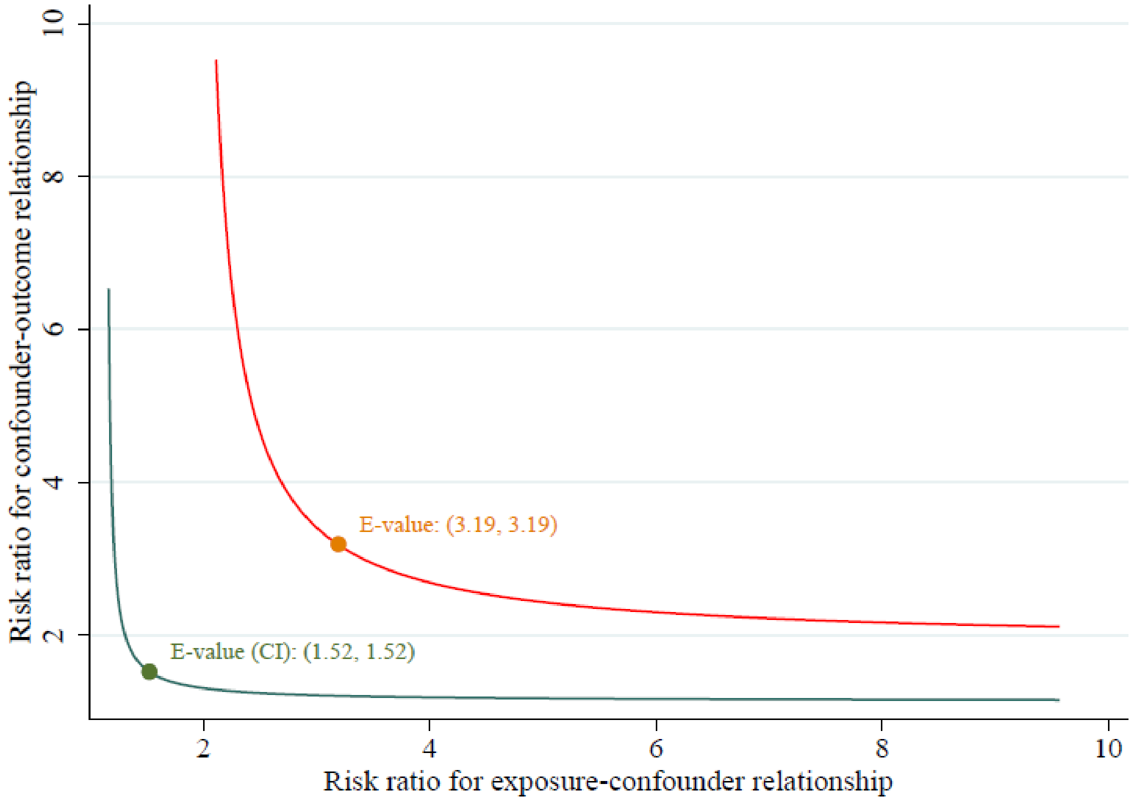

Figure S2: E-value for the lower 95% CI, point estimate of the atherogenic index of plasma, and incident heart failure. The value of the joint minimum strength of association on the risk ratio scale that an unmeasured confounder must have with the exposure and outcome to fully describe the AIP and HF hazard ratios.
